# Gender Differences Between Domestic Violent Men and Women: Criminogenic Risk Factors and Their Association With Treatment Dropout

**DOI:** 10.1177/08862605211063015

**Published:** 2021-12-29

**Authors:** Anne M. E. Bijlsma, Claudia E. van der Put, Annemiek Vial, Joan van Horn, Geertjan Overbeek, Mark Assink

**Affiliations:** 1 Research Institute Child Development and Education, University of Amsterdam, Amsterdam, The Netherlands; 2 Center for Outpatient Forensic Treatment, 84888 de Waag, Utrecht, Netherlands

**Keywords:** domestic violence, criminogenic risk factors, treatment dropout, gender differences, network analysis

## Abstract

Although many studies have concluded that men and women engage in domestic violence at equal levels, existing studies have hardly focused on gender specific risk factors for domestic violence perpetration. Therefore, this study aimed to examine gender differences in criminogenic risk factors between Dutch male and female forensic outpatients who were referred to forensic treatment for domestic violence. Clinical structured assessments of criminogenic risk factors were retrieved for 366 male and 87 female outpatients. Gender differences were not only found in the prevalence and interrelatedness of criminogenic risk factors, but also in associations between criminogenic risk factors and treatment dropout. In men, risk factors related to the criminal history, substance abuse, and criminal attitudes were more prevalent than in women, whereas risk factors related to education/work, finances, and the living environment were more prevalent in women. Further, having criminal friends, having a criminal history, and drug abuse were associated with treatment dropout in men, whereas a problematic relationship with family members, housing instability, a lack of personal support, and unemployment were associated with treatment dropout in women. Finally, network analyses revealed gender differences in risk factor interrelatedness. The results provide important insights into gender specific differences in criminogenic risk factors for domestic violence, which support clinical professionals in tailoring treatment to the specific needs of male and female perpetrators of domestic violence.

Domestic violence (defined as physical, sexual, emotional, economic, or psychological abuse against an intimate partner, child, or other relative) affects many men, women, and children (Carlson, 2000; Moylan et al., 2010; Tjaden & Thoennes, 2000; United Nations, 2020; Wolfe et al., 2003; World Health Organization, 2013). The devastating consequences of domestic violence ask for treatment programs with minimal dropout of perpetrators to reduce (recurring) family violence. Although women are more often portrayed as victims than perpetrators of domestic violence, recent studies report equal domestic violence victimization prevalence in men and women (de Vogel et al., 2016; Lysova et al., 2019). It is striking that even though a large part of the domestic violence perpetrators is female, not much is known about how female criminogenic risks differ from those of males, or which different criminogenic risk factors are associated with treatment dropout in females compared to males (de Vogel et al., 2014). Therefore, the aim of this study was to provide further insights into gender differences in forensic outpatients who were referred to forensic treatment for domestic violence, by studying gender differences in the prevalence of criminogenic risk factors, their interrelatedness using an innovative statistical technique for network modeling, and their association with treatment dropout.

Studies show that women experiencing intimate partner violence are at increased risk of experiencing physical and mental health problems, such as depression, trauma, and stress (e.g., Campbell & Lewandowski, 1997; Gorde et al., 2004). Since there is a general view in the literature that men are more often perpetrator than victim of domestic violence, there is also much less research on the consequences of domestic violence victimization for men (Archer, 2000; De Vogel & Uzieblo, 2020). However, there are studies available showing that a poor health, depressive symptoms, substance abuse, and injury, may follow domestic violence victimization of men (Coker et al., 2000; Randle & Graham, 2011). Besides the effects of domestic violence on the well-being of men and women alike, exposure to domestic violence is associated with externalizing and internalizing problems in children, such as increased aggressive behavior, trauma, and depression (e.g., Huth-Bocks et al., 2001; Evans et al., 2008; Jouriles et al., 2008). To reduce these consequences of family violence, effective treatment programs with minimal dropout of perpetrators are urgently needed.

Unfortunately, there is a lack of evaluation studies on the effects of intervention programs in female perpetrators (Carney et al., 2007). What we do know is that for male perpetrators, treatment effects for reducing domestic violence are small (e.g., *d* = 0.34, Babcock et al., 2002). A main cause of this disappointing finding can be found in high treatment dropout rates, as more than 40% of male perpetrators of domestic violence fail to complete treatment (Babcock et al., 2002; Buttell & Pike, 2002; Sartin et al., 2006). These high dropout rates are a major problem because treatment completion is necessary to sufficiently reduce the risk factors contributing to the likelihood of recidivism of perpetrators of domestic violence (Babcock & Steiner, 1999; Bennett et al., 2007; Rosenbaum et al., 2001; Jones et al., 2004). An important question is why these treatment attrition rates in interventions aimed at reducing domestic violence are so high.

Several studies addressed this question by identifying differences between dropouts and completers of domestic violence treatments. Results show that variables predictive of domestic violence treatment dropout correspond to variables that are predictive of criminal recidivism (Jewell & Wormith, 2010; Wormith & Olver, 2002). As Jewell and Wormith (2010) argue, many of the identified risk factors for treatment dropout reflect criminogenic needs from the Risk, Need, and Responsivity Model by Andrews and colleagues (1990). Criminogenic needs are dynamic risk factors that are directly linked to criminal behavior, such as mental health problems or coping skills. These risk factors can potentially be changed and therefore provide opportunities for treatment aimed at reducing criminogenic needs and strengthening protective factors (Babcock & Steiner, 1999; Bonta & Andrews, 2017; Olver et al., 2011; Tollefson et al., 2008). Daly and Pelowski (2000) also stressed that strategies for treatment retention include a thorough assessment of risk factors for treatment dropout, and close monitoring of perpetrators at higher risk for treatment attrition throughout their program participation.

Examples of dynamic criminogenic needs that reflect risk factors for treatment dropout in male perpetrators of domestic violence are psychological problems, unemployment, and substance abuse (Bowen & Gilchrist, 2006; Daly & Pelowski, 2000; Grusznski & Carrillo, 1988; Jewell & Wormith, 2010; Lila et al., 2017; Stalans & Seng, 2007; Tollefson et al., 2008). Besides criminogenic needs, static risk factors (i.e., immutable risk factors), such as a criminal history, or a history of victimization as a child, are also associated with treatment dropout in perpetrators of domestic violence, although conflicting results have been found (Daly & Pelowski, 2000; Grusznski & Carrillo, 1988; Jewell & Wormith, 2010; Rooney & Hanson, 2001; Scott, 2004). In the few studies on female perpetrators of domestic violence, quite similar risk factors for treatment dropout were found. For example, criminogenic needs (e.g., drug and alcohol use, unemployment, and low educational level) and static risks (e.g., criminal history) are associated with treatment attrition in both men and women (Buttell et al., 2012; Carney & Buttell, 2004).

Still, studies on criminogenic needs of perpetrators of domestic violence are primarily focused on men, and there are a limited number of studies on similarities and differences between male and female domestic violence perpetrators. One of those studies by Henning and colleagues (2003) showed that women arrested for domestic violence are more likely than men to have previously attempted suicide, and that they are more often previously treated with psychotropic medication (e.g., antipsychotics). On the contrary, male perpetrators are more often treated for substance abuse (Henning et al., 2003). Both male and female perpetrators show minimization, denial, and external attributions related to their domestic violent offense, but female perpetrators tend to attribute their violent offenses more often to characteristics of their partner, such as lack of commitment and unfaithfulness (Henning et al., 2005). Results from a study examining clinical and personality disorders diagnosed in male and female perpetrators of domestic violence showed that women demonstrated more histrionic, narcissistic, and compulsive personality traits compared to men (Simmons et al., 2005). This study also showed that men demonstrated higher dependent personality traits than women. Carney and colleagues (2007) argue that female perpetrators of domestic violence share similar motives and psycho-social characteristics (e.g., prior aggression or personality disturbance) as male perpetrators. Carney et al., (2007), also suggested that professionals would do well to consider common risk factors for general violence when evaluating possible intervention needs of male and female abusers. To date, no studies used comprehensive measures of criminogenic risk factors for criminal behavior and recidivism, such as risk factors forming the Central Eight (Bonta & Andrews, 2017; Eisenberg et al., 2019), in examining gender differences and similarities in male and female perpetrators of domestic violence.

Furthermore, while the risk for treatment dropout may increase by criminogenic risk factor interactions (Olver et al., 2011), no attention has been paid to risk factor interrelatedness in perpetrators of domestic violence. Advances in methodology and statistics have made it possible to study the complexity of the relations between risk factors, for example network analysis (Borsboom & Cramer, 2013). Using network analysis, partial correlations between risk factors can be examined, and the most central risk factor (i.e., the risk factor that is most likely to cause the development of other risks) can be determined (Borsboom & Cramer, 2013). This analysis provides important information for treatment directions, as it can be expected that targeting central risk factors in interventions helps reducing other risks.

Therefore, the aim of this study was to increase knowledge on gender specific criminogenic risk factors in forensic outpatients who were referred to forensic treatment for domestic violence by studying differences and interactions in risk factors between female and male forensic outpatients. More specifically, we examined gender differences in the prevalence of criminogenic risk factors, and examined the interrelatedness between the criminogenic risk factors in male and female outpatients using an innovative statistical technique for network modeling. Finally, we examined the association between the criminogenic risk factors and treatment dropout in both male and female outpatients. Because of a lack of substantial empirical attention to risk factors in female perpetrators of domestic violence, and inconsistencies in study results of risk factors for treatment dropout in male perpetrators of domestic violence, we were unable to develop specific hypotheses about differences in risk factors between these perpetrator groups. Yet, in light of the studies that are available, we did expect to find risk factors for treatment dropout that correspond to risk factors that are predictive of criminal recidivism (i.e., Central Eight criminogenic needs, Andrews et al., 1990) in both perpetrator groups (Jewell & Wormith, 2010; Wormith & Olver, 2002).

## Method

### Sample

The initial sample comprised 1272 adult forensic outpatients who were referred to forensic treatment for domestic violence between 2014 and 2015 at a forensic care facility in the Netherlands (de Waag). In this sample, 213 outpatients did not receive treatment because of various contraindications, such as acute psychosis and addiction. Data from another 204 outpatients were excluded, because of registration errors in the electronic files of these outpatients (e.g., information on the diagnostic phase was missing). Another 103 outpatients did not give permission for using their data for research purposes. Last, a complete risk assessment was not available for 752 outpatients implying that the final sample consisted of 453 outpatients (366 men and 87 women).

#### Demographics and treatment characteristics

Compared to the sampled women (*M* = 34.80, *SD* = 9.92), men were older (*M* = 38.58, *SD* = 11.18) (*t* (451) = 2.89, *p* < .01), more often court mandated (36% and 9%, respectively) (*x*^2^ (1, *N* = 453) = 22.99, *p* < .001), and more often had a non-Dutch nationality (32% and 22%, respectively) (*x*2 (1, *n* = 363) = 4.33, *p* < .05). There was no significant difference in treatment duration in months between men (*M* = 8.94, *SD* = 4.35) and women (*M* = 9.68, *SD* = 4.42) (*t* (351) = −1.21, *p* = .226).

### Research Protocol

The data used in this study were collected as part of routine outcome monitoring (ROM) at the forensic care facility (de Waag). This facility is the largest forensic outpatient treatment center in the Netherlands with approximately 5000 outpatients entering treatment each year. The facility offers mainly cognitive-behavioral based interventions to juvenile and adult outpatients who, due to their offensive behavior, come into contact with police force or judicial authorities. Patients enter treatment on a voluntary or mandatory basis. Voluntary treatment indicates that the patient enters treatment on his own initiative, either on referral of a general practitioner or another mental health care institute. Mandatory treatment means that treatment is imposed by a judge, and that a probation officer acts as supervisor.

The routine outcome monitoring (ROM) data in this study were collected by the therapists at the forensic care facility as part of their daily job activities, and were provided anonymously to the researchers. The ROM data collection is part of ongoing research at the forensic care facility that is aimed at improving regular treatment. In the ROM procedure, all outpatients referred to the facility are routinely assessed with a number of internet-based instruments (e.g., the Risk Assessment for outpatient Forensic Mental Health-Adult [RAF-MH]) at baseline during intake, and if treatment is initiated, repeatedly every four months during treatment. At intake, patients were informed by the therapist about what data will be collected and how their data will be used for scientific purposes. Patients were asked to sign a general informed consent letter if they agreed on the use of their data for scientific research, and they could withdraw their consent at any time during and after treatment. This procedure was in line with the Dutch Data Protection Act (Dutch DPA) and Dutch healthcare law that prescribe how the privacy of personal information in the context of mental health services must be dealt with.

#### Dropout

A premature ending of treatment by either the outpatient or the practitioner was referred to as dropout, which concerned 82 (22.4%) of the 366 male outpatients, and 21 (24.1%) of the 87 female outpatients in the sample (*x*^2^ (1, *N* = 453) = .120, *p* = .729). In the initial sample of outpatients for whom data of treatment completion were available, there was also no significant difference between the dropout rates of male (23.3%) and female outpatients (22.7%) (*x*^2^ (1, *N* = 544) = .015, *p* = .901). There were several reasons for dropping out of treatment, of which a persistent lack of motivation, frequent illicit absence from treatment sessions, or a lack of progress as assessed by the therapist, were the most common.

### Instruments

The Risk Assessment for outpatient Forensic Mental Health-Adult version (RAF-MH) is a structured professional judgment risk assessment instrument for adults for whom forensic psychiatric health care is indicated (van Horn et al., 2012). The RAF-MH consists of twelve so-called risk domains, each measuring at least of two or more criminogenic risk factors. The structure of the instrument is comparable to the Level of Service Inventory-Revised (LSI-R), which is a risk and needs assessment tool developed by Andrews and Bonta (2000). Similar to the LSI-R, the RAF-MH measures overall risk domain scores for criminal recidivism by assessing both static (e.g., age of onset for delinquent behavior) and dynamic (e.g., drug abuse) risk factors. Contrary to singular item scoring, this scoring structure offers the possibility of tracing the decision procedure that has resulted in overall risk domain scores. More specifically, this scoring structure enables a more explicit and clear risk assessment procedure than singular item scoring, particularly in retrieving the information that has led to the overall clinical judgment at the end of each risk domain. The risk assessment following the RAF-MH consists of two steps: (1) All risk domain items are scored by the therapist following the guidelines as described in the manual of the RAF-MH; (2) Each risk domain is given a structured clinical judgment on the overall functioning of the outpatient based on the underlying risk items. This judgment is expressed on a 6-point scale, with scores 0, 1, and 2 indicating a satisfactory level of functioning and with scores 3, 4, and 5 indicating a problematic level of functioning.

The 12 risk domains that can be assessed with the RAF-MH are: (1) “Previous and current offenses”: for example, previous criminal behavior and age at first antisocial behavior; (2) “School/(part-time) job”: for example, behavioral problems at school, or employment; (3) “Finances”: having debts and having an unemployment benefit; (4) “Living environment”: instability of living situation and living in a bad neighborhood; (5) “Family/partner”: for example, relationship instability and relationship with parents; (6) “Social network”: for example, social isolation and affiliation with deviant peers; (7) “Leisure activities”: individual- and group activities, (8) “Substances”: for example, substance abuse/dependency and its negative effect on several life domains; (9) “Emotional/personal”: for example, coping skills, impulsivity, and personality disorders; (10) “Attitudes”: for example, lack of empathy and crime supportive beliefs; (11) “Motivation for treatment”: for example, treatment attendance and insight in risky situations; Domain 12 “Sexual problems” only applies to sex offenders, and the scores in this domain were therefore excluded from the analyses.

The psychometric qualities of the adult version of the RAF-MH have not yet been examined, but the inter-rater reliability (Intraclass Correlation Coefficients (ICC) = 0.78) and predictive validity (Area Under the Curve (AUC) = 0.77) of the almost identical youth version of the RAF-MH are sufficient (Van Horn et al., 2009). A total of 21 items of the RAF-MH were scored dichotomously (no/yes), whereas 34 items were scored on a 3-point scale (ranging from 1–2–3) with higher scores indicating higher levels of a risk factor. For these non-dichotomous items, dummy variables were created, with 1 (score 1 or 2) indicating the presence and 0 the absence of a risk factor. One item from the risk domain “criminal history and severity,”, and seven items from the risk domain “education/work” were excluded from analyses, because the scores on these items were missing for more than 50% of the participants. Data were missing, for instance, because items were not applicable to a participant’s circumstances (e.g., job performance in case of unemployment).

### Analyses

A phi coefficient was computed by performing a Chi-Square test of independence to determine gender differences in the prevalence of criminogenic risk factors that were measured with the RAF-MH. An independent samples *t*-test was performed to determine gender differences in RAF-MH risk domain scores.

To examine the interrelatedness of the risk factors for male and female outpatients, statistical networks were created to model the interactions between risk domains. Network analysis is a relatively new method for modeling interactions between variables that is increasingly applied to different disciplines, for example, to explore the interrelatedness of risk factors for child maltreatment (Vial et al., 2020). A network characterizes structures in terms of nodes (the RAF-MH risk domains/factors) and edges (relationships or the partial correlations) that connect these nodes. We used the EBICglasso technique, which estimates partial correlations between all variables, and shrinks absolute weights to zero, addressing the multiplicity issue (Barbalat et al., 2019; Van den Bergh, 2018). Before interpreting the obtained networks, correlation stability (CS) coefficients were calculated to make inferences about the accuracy and stability of the node strength centrality and edge weight coefficients. The centrality measures and the edge weights are considered stable when the corresponding CS-coefficient exceeds a value of .25 (Epskamp et al., 2018). The network analyses were performed using R-package “bootnet” (version 1.2; Epskamp et al., 2018) in R-3.6.1). Correlation coefficients were interpreted using the guidelines by (Gignac & Szodorai, 2016) (i.e., 0.10 = small, 0.20 = moderate, and 0.30 = large).

For men and women separately, a phi coefficient was computed by performing a Chi-Square test of independence to determine the associations between the dichotomously scored variables: risk factors (present/not present) and treatment dropout (treatment dropout/treatment completion). The results were interpreted using the guidelines of Cohen (1988) (i.e., small = 0.1, moderate = 0.3, and large = 0.5). For every risk factor item, a two-proportion *z*-test was performed to determine gender differences in risk prevalence in dropouts. For every domain, bivariate correlation analyses were conducted to determine the association between risk factor scores and dropout. The effect sizes were interpreted using the guidelines of Rice and Harris (2005) for point-biserial correlations (i.e., men: small = 0.081, moderate = 0.204, large = 0.316, women: small = 0.085, moderate = 0.209, large = 0.324). A comparison of correlations from independent samples (*z*-test) was performed to determine significant gender differences in association strength between domain risk scores and treatment dropout (Lenhard & Lenhard, 2014).

## Results

### Risk Factor Prevalence and Risk Domain Scores

Table 1 provides an overview of the prevalence of criminogenic risk factors (in percentages) for male and female outpatients as measured by the RAF-MH. Fifteen risk factors were significantly more prevalent in male than in female outpatients, of which six were static (i.e., prior convictions, official offense records, unreported offenses, previous imprisonment, past alcohol abuse/dependence, and past drug abuse/dependence), and nine were dynamic (i.e., criminal friends, present alcohol abuse/dependence, present drug abuse/dependence, substance use disorder, interpersonal problems because of substance use, poor anger management, offense justification, offense denial, and lack of empathy). In female outpatients, one static risk factor (i.e., victim of child maltreatment) and four dynamic risk factors (i.e., currently unemployed, low job performance, unemployment benefit, and housing instability) were significantly more prevalent than in male outpatients. Table 2 provides the mean scores on the RAF-MH risk domains for male and female outpatients. Men scored significantly higher than women on criminal history, substance abuse, and criminal attitudes. Both male and female outpatients scored high on the personal/emotional risk domain.

**Table 1.** Prevalence of Criminogenic Risk Factors in Domestic Violent Men and Women.

Risk Factor	Risk Prevalence		
	*N* Men/Women	(%) Men/Women	*r*_φ_^a^
1. Criminal history			
Convictions	366/87	52/23	−.231***
Official offense records	360/87	48/24	−.193***
Unreported offenses	315/83	73/59	−.121*
Weapon use/threat of death	344/84	26/23	−.032
Offense frequency/severity	348/84	54/44	−.081
Age of onset of delinquent behavior	330/60	32/27	−.042
Previous imprisonment	349/87	20/6	−.154***
2. Education/Work			
Problematic employment history	343/80	49/40	−.068
Currently unemployed	363/85	39/57	.142**
Job performance	208/33	5/15	.147*
3. Finances			
Unemployment benefit	361/85	35/55	.162***
Debt	346/80	51/51	.003
4. Living environment			
Housing stability	363/86	28/42	.123**
Disadvantaged neighborhood	316/80	24/28	.032
5. Family/Spouse			
Relationship instability	360/87	94/95	.017
Relationship with caregivers	339/83	53/60	.055
Relationship with family members (and in-laws)	328/73	65/66	.007
Relationship with children	277/66	41/35	−.051
Family members with police contacts	283/60	18/18	.000
6. Social Environment			
Social isolation	351/83	22/19	−.023
Criminal friends	312/70	24/13	−.107*
Availability of personal support	348/86	56/61	.036
7. Leisure activities			
Individual leisure activities	326/76	61/68	.062
Contextual leisure activities	320/79	73/72	−.012
8. Substance abuse			
Alcohol abuse/dependence (past)	349/82	41/13	−.226***
Drug abuse/dependence (past)	353/82	37/23	−.113*
Alcohol abuse/dependence (present)	342/85	21/8	−.127**
Drug abuse/dependence (present)	348/84	24/7	−.163***
Substance use disorder	351/85	41/15	−.208***
Interpersonal problems because of substance use	337/80	36/18	−.155**
School/work problems because of substance use	322/79	7/3	−.076
9. Personal/Emotional			
Victim of child maltreatment	330/77	42/57	.123*
Bullied in school	276/63	25/32	.056
Suicidal thoughts	335/78	30/29	−.003
Lack of self-insight	356/85	82/74	−.076
Impulsivity	356/84	84/79	−.060
Stress factors	363/85	96/100	.084
Coping skills	360/84	63/54	−.079
Anger management	355/82	98/87	−.203***
Axis I diagnose	332/81	80/80	.004
Axis II diagnose	342/82	41/45	.031
Cognitive impairments	352/82	15/20	.044
10. Criminal attitudes			
Offense justification	356/84	43/29	−.118*
Offense denial	357/86	49/29	−.161***
Lack of empathy	354/81	58/33	−.190***
11. Treatment engagement			
Health care history	215/62	50/53	.029
Treatment motivation	360/86	35/28	−.061

^*^ *p* <.05.

^**^ *p* <.01.

^***^ *p* <.001.

a Strength of the association (*r*_φ_) between gender (man/women) and risk item prevalence. Gender was scored dichotomously (0 = men, 1 = women), meaning that a negative phi coefficient indicates a higher risk factor prevalence in men.

**Table 2.** Gender Differences in Risk Domain Scores.

	Risk domain scores *M* (*SD*)		
Risk domain	Men (*n* = 358)	Women (*n* = 85)	*t*^a^
1. Criminal history	3.03 (1.11)	2.10 (1.53)	5.31***
2. Education/Work	1.98 (1.46)	2.06 (1.26)	−.50
3. Finances	1.83 (1.51)	2.00 (1.47)	−.97
4. Living environment	1.18 (1.33)	1.34 (1.20)	−1.05
5. Family/Spouse	3.40 (0.96)	3.42 (1.07)	−.17
6. Social environment	1.94 (1.37)	1.92 (1.34)	.12
7. Leisure activities	1.67 (1.39)	2.00 (1.35)	−1.95
8. Substance abuse	1.74 (1.66)	0.81 (1.38)	5.37***
9. Personal/Emotional	3.44 (0.92)	3.54 (1.02)	−.81
10. Criminal attitudes	1.87 (1.35)	1.31 (1.32)	3.47***
11. Treatment engagement	1.52 (1.36)	1.42 (1.32)	.57

^*^ *p* <.05.

^**^ *p* <.01.

^***^ *p* <.001.

^a^An independent samples *t*-test was performed for each risk domain to test the difference in mean domain risk score between male and female domestic violence offenders. Gender was scored dichotomously (0 = men, 1 = women), meaning that a negative *t* value indicates a higher risk domain score in men.

### Risk Domain Interrelatedness

**Figure 1.**
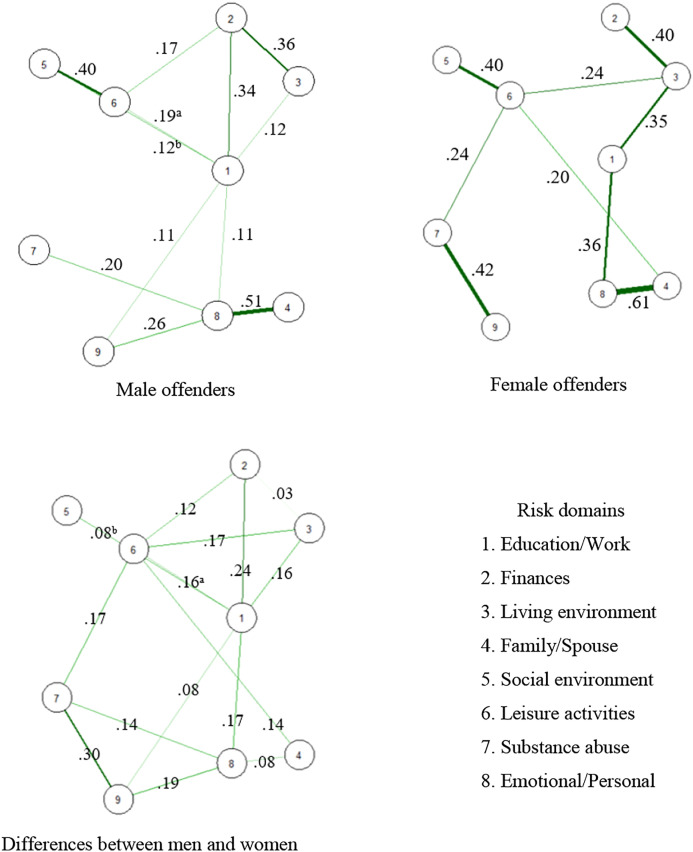
Networks of risk domains for men and women. Note. The networks depict the interrelatedness of the risk domains in male and female domestic violent perpetrators. ^a^Correlation between risk domains 1 and 5; ^b^Correlation between risk domains 1 and 6.

**Figure 2.**
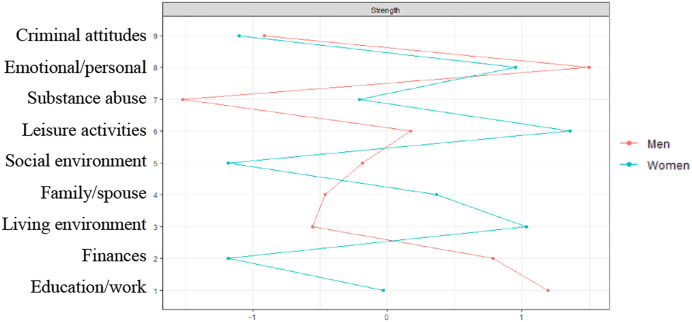
Network centrality of risk domains for men and women. *Note.* Standardized strength centrality coefficients (z-Scores, *x*-axis). A higher z-score indicates that a node (risk domain) is more influential in the network, based on the strength of the connections with other risk domains. For men, the risk domains “emotional/personal” and “education/work” were the most central in the risk domain network. In both networks, all risk domains were positively correlated. The strength centrality in the risk domain network of women was not sufficiently stable, and could therefore not be interpreted.

Figures 1 and 2 show the results of the network analyses that were performed to examine the interrelatedness of the risk domains in male and female outpatients. The network for male outpatients was sufficiently stable, as the CS-coefficients of the strength centrality and edge weight were .28 and .60. For female outpatients, the edge weights (i.e., partial correlation coefficients) were sufficiently stable (.29) according to the criteria of Epskamp et al. (2018), but the overall strength centrality coefficient was below the preferred value of .25, meaning that the risk domain centrality could not be interpreted. For male outpatients, the risk domains “emotional/personal” and “education/work” play the most central role in the risk domain network (Figure 2). In both networks, all risk domains were positively correlated. The strength of all correlations can be found in Figure 1. For both male and female outpatients, the strongest relation in the network was found between the risk domains “family/spouse” and “emotional/personal” (Figure 1). Further, moderate relations were found between the risk domains “social environment” and “leisure activities,” and between “finances” and “living environment.” There were also differences between male and female outpatients in risk domain interrelatedness. In female outpatients, the “education/work” domain was moderately associated with both “living environment” and “emotional/personal,” and the “substance abuse” domain was moderately associated with “criminal attitudes.” In male outpatients, a moderate relation was only found between “education/work” and “finances.”

### Risk Factors and Treatment Dropout

Table 3 reveals how the criminogenic risk factors assessed with the RAF-MH are related to treatment dropout in male and female outpatients. For male outpatients, small positive significant effect sizes were found for 16 items, which could be designated as risk factors for treatment dropout (e.g., previous imprisonment, problematic employment history, debt, family members with police contacts, having criminal friends, drug/alcohol abuse/dependence, offense justification, lack of empathy, and insufficient treatment motivation). For female outpatients, a problematic relationship with family members (and in-laws) was identified as risk factor for treatment dropout with a significant moderate positive effect size. Furthermore, significant small positive effect sizes were found for three identified risk factors for treatment dropout in female outpatients: unemployment, housing instability, and lack of personal support. The factors criminal friends and substance use disorder were significantly stronger related to treatment dropout in male outpatients, whereas the factor problematic relationship with family members (and in-laws) was significantly stronger related to dropout in female outpatients.

**Table 3.** Associations between Criminogenic Risk Factors and Treatment Dropout in Domestic Violent Men and Women.

Risk Factor	Dropout		
	Men *r*_φ_^a^	Women *r*_φ_^a^	*z*^b^
1. Criminal history			
Convictions	.055	.139	−.70
Official offense records	.018	−.067	.70
Unreported offenses	.119*	−.013	1.06
Weapon use/threat of death	.047	.082	−.28
Offense frequency/severity	.010	−.069	.64
Age of onset of delinquent behavior	.193***	.140	.38
Previous imprisonment	.173***	−.024	1.63
2. Education/Work			
Problematic employment history	.133*	.049	.67
Currently unemployed	.100	.228*	−1.08
Job performance	.055	—	—
3. Finances			
Unemployment benefit	.104*	.164	−.50
Debt	.128*	.159	−.25
4. Living environment			
Housing stability	.095	.286**	−1.63
Disadvantaged neighborhood	.034	−.064	.77
5. Family/Spouse			
Relationship instability	.044	−.004	.44
Relationship with caregivers	.060	.055	.04
Relationship with family members (and in-laws)	−.022	.379**	−3.19***
Relationship with children	.043	.106	-.45
Family members with police contacts	.161**	−.002	1.13
6. Social Environment			
Social isolation	-.018	.082	−.81
Criminal friends	.292***	−.074	2.78**
Availability of personal support	.046	.238*	−1.61
7. Leisure activities			
Individual leisure activities	.057	.131	−.58
Contextual leisure activities	.062	.085	−.18
8. Substance abuse			
Alcohol abuse/dependence (past)	.098	.123	−.20
Drug abuse/dependence (past)	.168**	.178	−.08
Alcohol abuse/dependence (present)	.130*	−.065	1.59
Drug abuse/dependence (present)	.205***	.062	.12
Substance use disorder	.161**	−.082	1.99*
Interpersonal problems because of substance use	.111*	−.159	2.15*
School/work problems because of substance use	.058	−.088	1.15
9. Personal/Emotional			
Victim of child maltreatment	.089	.044	.35
Bullied in school	-.041	.179	−1.56
Suicidal thoughts	.010	−.039	.38
Lack of self-insight	.063	.027	.29
Impulsivity	.082	.101	−.16
Stress factors	.033	—	—
Coping skills	.018	-.014	.26
Anger management	.042	.057	−.12
Axis I diagnose	.082	−.075	1.25
Axis II diagnose	.050	.029	−.23
Cognitive impairments	.077	.079	−.02
10. Criminal attitudes			
Offense justification	.108*	.080	.23
Offense denial	.014	.133	−.98
Lack of empathy	.108*	.020	.71
11. Treatment engagement			
Health care history	.105	.077	.19
Treatment motivation	.120*	.008	.92

^a^Strength of the association (*r*_φ_) between treatment dropout and risk item prevalence.

^b^Gender differences (*z*) in risk prevalence in dropouts.

The associations between risk domain scores and treatment dropout are shown in Table 4. For male dropouts, small positive effect sizes were found for nine risk domains, of which six were significant and therefore designated as risk domains for treatment dropout: education/work, finances, living environment, social environment, substance abuse, and treatment engagement. The risk domain substance abuse was significantly stronger related to treatment dropout in male than in female outpatients.

**Table 4.** Gender Differences in the Association between Risk Domain Scores and Treatment Dropout.

	Relation to dropout (*r*_*pb*_)^a^		
Risk domain	Men (*N* = 358)	Women (*N* = 85)	*z*^b^
1. Criminal history	.094	−.038	1.07
2. Education/Work	.157**	.060	−.16
3. Finances	.146**	.073	−.17
4. Living environment	.181***	.133	−.66
5. Family/Spouse	.046	−.100	.90
6. Social environment	.134*	.035	−.41
7. Leisure activities	.071	.061	.25
8. Substance abuse	.150**	−.022	3.64***
9. Personal/Emotional	.099	−.038	.97
10. Criminal attitudes	.084	.111	.42
11. Treatment engagement	.113*	−.039	.57

^*^ *p* <.05.

^**^ *p* <.01.

^***^ *p* <.001.

^a^Strength of the association (*r*_*pb*_)between risk domain scores and treatment dropout.

^b^Gender difference (*z*) in correlation between domain risk score and treatment dropout.

## Discussion

The aim of this study was to increase knowledge on differences in criminogenic risk factors between female and male forensic outpatients who were referred to forensic treatment for domestic violence. The results revealed important gender similarities and differences with regard to the prevalence of criminogenic risk factors, the interrelatedness of criminogenic risks, and the extent to which those factors were associated with treatment dropout. The most important results are discussed below.

The risk factors with the highest prevalence in both male and female forensic outpatients were emotional and personal risk factors (e.g., lack of self-insight, stress factors, impulsivity, and anger management). These results are in line with previous findings of the presence of (negative) emotional factors, such as anger and hostility, in domestic violence of both male and female perpetrators (Birkley & Eckhardt, 2015). An important gender difference emerged as well: socioeconomic risk factors (e.g., unemployment and housing instability) were more prevalent among female outpatients than male outpatients. These results support previous findings of female perpetrators being disproportionately affected by poverty and related social policies (Holtfreter & Wattanaporn, 2014). Alcohol and drug abuse were more prevalent in men than in women, which is consistent with the finding that male perpetrators of domestic violence are more often treated for substance abuse than female perpetrators (Henning et al., 2003). Also, in accordance with previous findings, our study showed that male outpatients more often had a criminal history (e.g., official offense records and previous imprisonment) and showed more criminal attitudes (e.g., offense justification, offense denial, and lack of empathy) than female outpatients (Rebecca Block et al., 2010; De Vogel & de Spa, 2019; Henning et al., 2003).

In a next set of network analyses, we showed that contrary to the network of male outpatients, substance abuse was strongly related to criminal attitudes in the network of female outpatients. Since risk factor interaction can cause an increased risk for treatment attrition, targeting criminogenic risk factors that are closely related to other risks may be an important strategy in increasing treatment retention (Olver et al., 2011). Network analysis in this study revealed a central position of the emotional/personal risk domain in the interrelatedness to other risk domains in both male and female outpatients. This means that targeting this domain in treatment could reduce other factors that are related to emotional/personal criminogenic risk factors (Barbalat et al., 2019). For example, risk factors belonging to the family/spouse risk domain, to which the emotional/personal risk domain was related in the networks of male and female outpatients. The overall strength centrality coefficient in the network for female outpatients was not sufficiently stable, and could therefore not be interpreted. Future, larger scale studies on risk factor interaction are recommended to provide further insights into prioritizing treatment goals in female perpetrators of domestic violence.

Third, we determined the associations between criminogenic risk factors and treatment dropout in both male and female forensic outpatients. Consistent with previous findings, alcohol and drug abuse and having a criminal history and criminal friends were positively associated with treatment dropout in male perpetrators of domestic violence (e.g., Bowen & Gilchrist, 2006; Henning et al., 2003; Jewell & Wormith, 2010). In female outpatients, unemployment, housing instability, having an unstable relationship with family members (and in-laws), and a lack of personal support were identified as treatment dropout risk factors. These results are in line with findings by Buttell and colleagues (2012), indicating that treatment attrition or completion in female perpetrators of domestic violence basically depends on socioeconomic risks and supports during program participation.

### Strengths and Limitations

To our knowledge, no studies used comprehensive measures of criminogenic risk factors for criminal behavior and recidivism in examining gender differences and similarities in domestic violence perpetrators. This study was the first to address this gap by identifying risk factors for treatment dropout and risk factor interrelatedness specifically in samples of male and female forensic outpatients who were referred to forensic treatment for domestic violence. Further, this study used an innovative statistical technique for network modeling. However, it is important to acknowledge some limitations.

First, the psychometric qualities of the adult version of the RAF-MH have not yet been examined, and therefore, predictive performance of the instrument on treatment dropout should be addressed in further research. However, the instrument includes the Central Eight criminogenic needs (Bonta & Andrews, 2017), that reflect well-established risk factors for criminal recidivism corresponding to risk factors for treatment dropout (Jewell & Wormith, 2010). Second, the relatively small number of females in our sample have negatively affected the statistical power in the analyses. However, even for the relatively small sample of female forensic outpatients (*n* = 87), the statistical power to detect a significant medium sized effect is 83%, which can be considered sufficient. Although many risk factors were significantly associated with treatment dropout, most effect sizes were small, meaning that the external validity of the findings should still be interpreted with caution. Third, reasons for treatment attrition, such as a lack of motivation, or a lack of progress, were not specifically registered for each outpatient. This information could be useful in analyzing more specific associations between risk factors and reasons for treatment dropout in further research. Fourth, there were significant differences in demographics between the sampled male and female outpatients (i.e., age, ethnicity, and the likelihood of being court mandated to treatment). These variables may have affected the results in this study, for example because the severity and impact of dynamic risk factors may vary across age groups (Spruit et al., 2017). Further research should be undertaken to examine possible interactions between such demographic variables. Fifth, the data used in this study concerned retrospective file data that were collected as part of a ROM procedure at the forensic care facility, meaning that the instrument (i.e., RAF-MH) used has not been preselected by the researchers. However, this generic structured professional judgment instrument has been based on well-known risk factors for recidivism, and fits the circumstances of clients referred to Dutch forensic outpatient treatment specifically (Wilpert et al., 2018). It was therefore considered as an appropriate measure to meet the aims of this study. Last, factors that predict general recidivism may not be the same for men and women, and there is an ongoing debate on whether risk assessment tools are sufficiently gender responsive (de Vogel et al., 2019; Henning et al., 2009). Broadening risk assessment by measuring unique needs of female perpetrators, such as abuse and trauma, self-esteem and assertiveness, and parenting and child care, in risk assessment instruments for perpetrators of domestic violence may contribute to further insights into gender differences in risk factors for criminal recidivism (Hollin & Palmer, 2006).

### Clinical Implications

An important strategy in reducing high treatment attrition rates among male and female perpetrators of domestic violence is identifying those clients who are at risk of dropping out through risk factor assessment (Daly & Pelowski, 2000). The results in this study indicated that a detailed, structured risk assessment designed for predicting criminal recidivism can support care providers in identifying risk factors for treatment dropout in an early treatment stage. At the same time, the results showed that highly prevalent criminogenic risk factors in perpetrators are not necessarily associated with treatment dropout. Thus, just because a criminogenic factor is highly *prevalent* in a risk population, this does not necessarily make it the most *relevant* target for boosting participation and intervention uptake. It should be noted that an attrition profile for perpetrators should be avoided, as this could undermine their chances for success in treatment (Olver et al., 2011). Rather, awareness of the presence of factors that contribute to the risk of treatment dropout should lead to increasing efforts to retain those clients who are most likely to drop out of treatment.

Despite much evidence that undermines the gendered perspective of domestic violence (i.e., the belief that men are more often perpetrators than women), this approach is often reflected in the aims of many organizations to date (Dixon & Graham-Kevan, 2011; Dutton, 2007). In addition, women convicted of domestic violence offenses are still often mandated into batterer intervention programs designed to intervene with male perpetrators (Carney et al., 2007). Gender inclusive policy is necessary to encourage professionals to be open to the idea that men and women can be both perpetrators and/or victims of domestic violence (Dixon & Graham-Kevan, 2011). Many of the identified risk factors for treatment dropout in this study reflect dynamic criminogenic needs and responsivity factors (e.g., criminal attitudes, criminal friends, alcohol abuse, housing instability, and lack of personal support) (Bonta & Andrews, 2017). By providing gender sensitive interventions that are tailored to those criminogenic needs, the risk of dropping out may be reduced.

Specifically, this study emphasizes the importance of providing socioeconomic support and resources to female perpetrators of domestic violence, which may increase treatment completion and thereby treatment effectiveness in reducing domestic violence perpetrated by women (Buttell et al., 2012). For example, providing state-sponsored resources to address short-term needs (e.g., housing stability), may substantially reduce the odds of recidivism in women perpetrators (Holtfreter & Wattanaporn, 2014). Further, in preventing reoffending, providing vocational and educational training to female perpetrators is essential for obtaining jobs that provide a living wage when they re-enter society (Shearer, 2003).

Further, this study emphasizes the importance of providing substance-abuse treatment as a component of an overall intervention for specifically male perpetrators of domestic violence (Fals-Stewart & Kennedy, 2005; Hirschel et al., 2010). Although treating alcohol use is proven to be an effective approach for reducing domestic violence, this is not a common strategy yet (Klostermann, 2006). In addition, substance abuse treatment programs should address domestic violence in terms of strengthening referral to other care providers, or developing expertise among their own program staff (Klostermann, 2006). This dual treatment may be an expensive investment, but the social and psychological costs of continued domestic violence are likely to be far higher (Hirschel et al., 2010).
